# The influence of two photobiomodulation protocols on orthodontically induced inflammatory root resorption (a randomized controlled clinical trial)

**DOI:** 10.1186/s12903-022-02251-w

**Published:** 2022-06-05

**Authors:** Farah Y. Eid, Walid A. El-Kenany, Mohamed I. Mowafy, Ahmed R. El-Kalza

**Affiliations:** Department of Orthodontics, Faculty of Dentistry, Alexandria University, Champolion Street, Azarita, Alexandria, Egypt

**Keywords:** Photobiomodulation, Laser, Orthodontically induced inflammatory root resorption, Cone-beam computed tomography

## Abstract

**Background:**

Controversial results have been reported regarding the impact of photobiomodulation (PBM) on orthodontically induced inflammatory root resorption (OIIRR). The aim of this study was to evaluate the influence of two PBM protocols, one of them requiring a high application frequency (on days 0, 3, 7, 14, then every 2 weeks), while the second requires less frequent applications (every 3 weeks), on OIIRR accompanying orthodontic treatment.

**Methods:**

Twenty female patients were recruited for this randomized controlled trial, requiring the therapeutic extraction of maxillary first premolars, and they were randomly divided into 2 equal groups. In Group A, one side of the maxillary arch randomly received PBM on days 0, 3, 7, 14, and every 2 weeks thereafter, while in Group B, one side was randomly chosen to receive PBM every 3 weeks. The laser applied was a Diode laser with a wavelength of 980 nm, in a continuous mode. Canine retraction in both groups was carried out using closed-coil springs, delivering 150 g of force, and the force level was checked every 3 weeks, over a 12-week study period. Pre-retraction and post-retraction cone-beam computed tomography (CBCT) was done for the evaluation of OIIRR.

**Results:**

No significant differences in the amount of OIIRR have been reported between the laser and control sides in both groups A and B. Also, no significant differences have been reported between the laser sides in both groups.

**Conclusions:**

Photobiomodulation does not affect OIIRR, whether by increasing or decreasing its occurrence, with both laser application protocols. Therefore, it can be stated that PBM does not result in root resorption less than the commonly observed range elicited with conventional orthodontic treatment, and that it has no effect on OIIRR.

*Trial registration* Two Low-level Laser Irradiation Protocols on the Rate of Canine Retraction (NCT04926389), 15/06/2021—retrospectively registered. https://clinicaltrials.gov/ct2/show/NCT04926389.

## Background

Orthodontically induced inflammatory root resorption (OIIRR) is known to be a common iatrogenic outcome of orthodontic treatment. However, despite its relative mildness in most of the cases, it is very frustrating when perceived radiographically. It has been agreed that orthodontic force application brings about a local inflammatory response in the surrounding periodontium, and this inflammation is considered the main reason behind the root resorption process [1]. Several authors proposed that OIIRR could be a side-effect of the cellular response accompanying the elimination of the hyalinized zone of the PDL, and simultaneously the removal of the anti-resorptive cementoid layer [2, 3].

The etiologic or risk factors contributing to OIIRR are multifactorial, caused by a blend of biological factors, in addition to the mechanical characteristics of the employed orthodontic forces [4]. Biological factors include genetic predisposition [5], age [6], root morphology [6], and pre-existing root resorption [7]. Factors related to orthodontic treatment mechanics include the force magnitude [8, 9], the overall treatment duration [10, 11], and the manner of force application [8].

Different procedures have been suggested, aiming to reduce the occurrence of OIIRR, or to potentially repair it. These suggestions included several drugs, such as steroidal and non-steroidal drugs [12], fluoride [13], calcitonin [14], and tetracycline [15]. However, other non-invasive adjuncts that were also clinically suitable, have been proposed for the same purpose, such as Photobiomodulation (PBM) [16, 17].

In contrast to the injected chemicals or the orally ingested drugs, PBM does not generate any systemic consequences, which might influence the general health of the patient [18]. PBM is also known to have a biostimulatory effect when applied to the target tissue areas, characterized by the enhancement of the bone remodelling process [16]. Moreover, as a consequence to the reparative and anti-inflammatory effects of PBM [19], it has been suggested that it may help in the reduction, the prevention, or the repair of root resorption [16, 17, 20, 21]. However, several studies opposed this reported effect for PBM, and according to their results, laser administration had no perceivable effect on OIIRR [22–24]. This divergence in the documented outcomes is probably attributed to the different laser application protocols, wavelength, output power, irradiation time, energy density, treatment interval, and so on, making direct comparisons between different studies rather difficult.

Several PBM application protocols have been reported in the literature, and some of them were found to require a high frequency of patient recall which is considered a major downside, such as that involving laser exposures on days 0, 3, 7, 14, and then every 2 weeks [25–27]. On the other hand, other protocols have been proposed with laser irradiations performed less frequently, making it more convenient to patients, such as that involving LLLT application every 3 weeks [28–30].

Therefore, the purpose of this study was to evaluate the influence of two different PBM protocols on OIIRR accompanying orthodontic tooth movement (OTM), one of them requiring a high laser application frequency (on days 0, 3, 7, 14, and every 2 weeks thereafter), while the second protocol requires less frequent applications (every 3 weeks), and thus less patient recall visits.

## Materials and methods

### Study design

The study was a randomized controlled clinical trial, involving two parallel groups, each evaluating one of the tested PBM application protocols. Each group employed the split-mouth design, with one side serving as the control group, and the other side serving as the study group.

### Study subjects

Twenty female patients requiring the extraction of maxillary first premolars as a part of their orthodontic treatment with subsequent canine retraction have been recruited for the study, with an age range from 15 to 20 years. The sample size was calculated based on an alpha error of 5%, and an 80% study power. This calculation was based on the mean and standard deviation of canine retraction in the study by Doshi-Mehta and Bhad-Patil [31], regarding LLLT application on days 0, 3, 7, 14, and then every 2 weeks (Group A), and those in the study by Qamruddin et al. [28] regarding LLLT application every 3 weeks (Group B). Ethical approval was attained from the Institutional Review Board of the Faculty of Dentistry, Alexandria University, Alexandria, Egypt (IRB: 00010556-IORG: 0008839). Manuscript Ethics Committee number 0111-01/2020. Patient recruitment was done from the outpatient clinic, Department of Orthodontics, Faculty of Dentistry, Alexandria University. Subjects were examined and screened, with the following eligibility criteria being considered: healthy systemic condition with no chronic diseases, no previous orthodontic treatment, adequate oral hygiene, and a healthy periodontium. All patients were informed of the procedure and signed informed consents accordingly. All research procedures were performed in accordance with the relevant guidelines and regulations, as stated in the Declaration of Helsinki.

### Patient preparation

The enrolled subjects were prepared for fixed orthodontic treatment by recording their medical and dental history, in addition to taking routine orthodontic records (intra-oral and extra-oral photographs, X-rays, and dental models). Reinforcement of oral hygiene measures was also ensured before the onset of orthodontic treatment. Maxillary and mandibular straight wire fixed Roth appliances were bonded, with 0.022 $\times$ 0.028 inch slots (Mini 2000; Ormco, USA) in all patients, followed by their referral for maxillary first premolars’ extraction. Leveling and alignment was then started and was considered complete when a 0.016 $\times$ 0.022 inch stainless steel arch wire could be placed passively in all the maxillary teeth.

### Randomization and patient allocation

Before the onset of canine retraction, all 20 patients were randomly assigned to either Group A or Group B (10 in each group), for laser administration. Randomization was done using a simple randomization process with an allocation ratio of 1:1. A box was arranged containing 20 folded pieces of paper, 10 of which had the word “Group A” written on them, while the other 10 papers had the word “Group B”. Each participant was asked to choose one of the folded pieces of paper from the box, and accordingly was assigned to one of the two groups. The same procedure was repeated once again within each group to assign one side of the maxillary arch to be the “study”, with the contralateral side serving as the “control” in the split-mouth design. Canine retraction in groups A and B, on both the experimental and the control sides was performed using nickel-titanium (NiTi) closed-coil springs, stretched between the canine bracket hook and the hook on the molar tube, delivering a force of 150 g as measured by a force gauge. The applied force was checked every 3 weeks, with each follow-up visit. Pre-retraction and post-retraction cone beam computed tomography (CBCT) was performed by all the enrolled subjects.

### Laser application

**Fig. 1:**
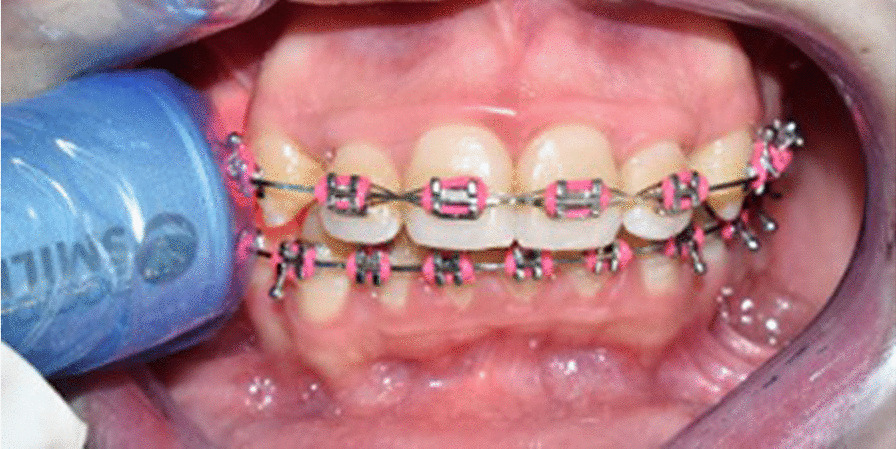
Optical fiber tip held against the maxillary canine root on the experimental side, at a distance of 1.5 cm, as per manufacturer instructions

The administered PBM was a Diode laser (Wiser; Doctor Smile-Lambda Spa, Brendola, Italy), emitting infrared radiation at a wavelength of 980 nm, and a power output of 100 mW, in a continuous mode. The plane wave optical fiber (AB 2799; Doctor Smile-Lambda Spa, Brendola, Italy) dispensed a beam spot size of 1 cm^2^ using the flat top handpiece, and the irradiation was performed on the experimental side by placing the optical fiber tip along the maxillary arch against the middle third of the canine root, where the irradiation would also reach the apical and the cervical thirds. The flat top handpiece was held at a distance of 1.5 cm as minimum on defocalization, as per manufacturer instructions, for 8 s (Fig. 1). The total energy density conducted per episode was 8 J/cm^2^ (1 J/cm^2^ per second). Precautions were taken prior to laser application, where both the patient and the operator used protective eyeglasses supplied by the manufacturer, specific for the employed wavelength.

**Fig. 2:**
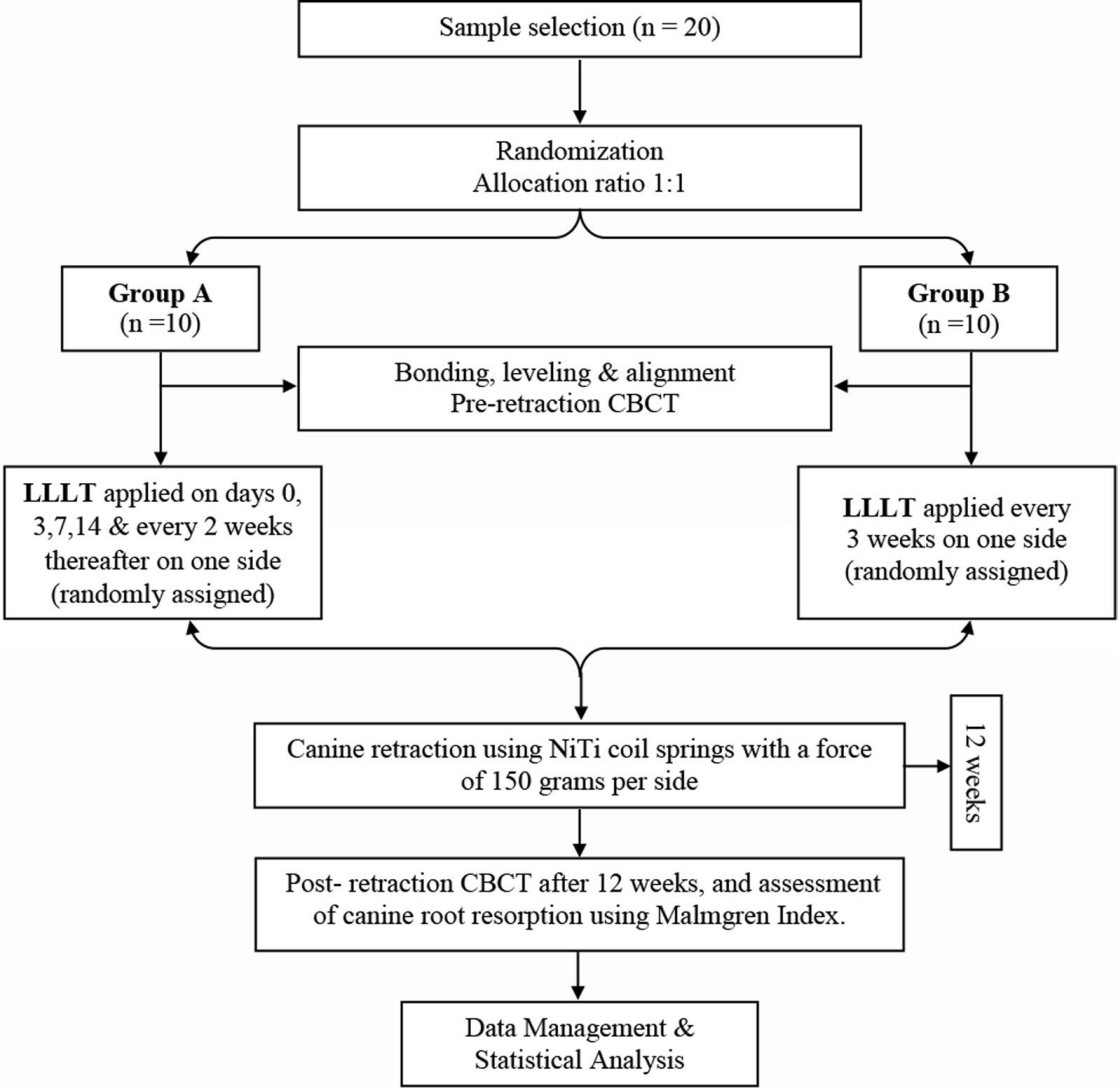
Research design flowchart

In Group A, subjects received PBM on days 0, 3, 7, 14, and every 2 weeks thereafter, whereas in Group B, PBM was applied every 3 weeks on the experimental sides throughout the study period, which was 12 weeks. The laser beam was also held passively on the control sides of both groups, providing a placebo effect, as a part of the blinding process for the enrolled patients. A research design flowchart is represented in Fig. 2, summarizing the study procedures.

### Canine root resorption measurement

Root resorption of the maxillary canines was evaluated and measured on the acquired pre-retraction and post-retraction CBCT scans, that were performed using the same + CBCT machine (J. Morita R100 Cone beam 3D Imaging System; MFG Corp., Kyoto, Japan). The scan was done with a Field of View (FOV) of 100 × 50 mm (Width × Height). The volumes were reconstructed with a 0.160 mm isometric voxel size. Also, the tube voltage was 90 kVp and 8 mA, whereas the exposure time was 20 s.

For the assessment of root resorption, the index suggested by Malmgren et al. [32] was used, where they constructed a classification for the evaluation of root resorption consisting of 5 grades; 0: No root resorption. 1: Mild resorption, normal root length but irregular contour. 2: Moderate resorption, apical root resorption (under 2 mm of the initial root length). 3: Severe resorption, apical root resorption (2 mm to one third of the initial root length). 4: Extreme resorption (exceeding one third of the root length).

**Fig. 3:**
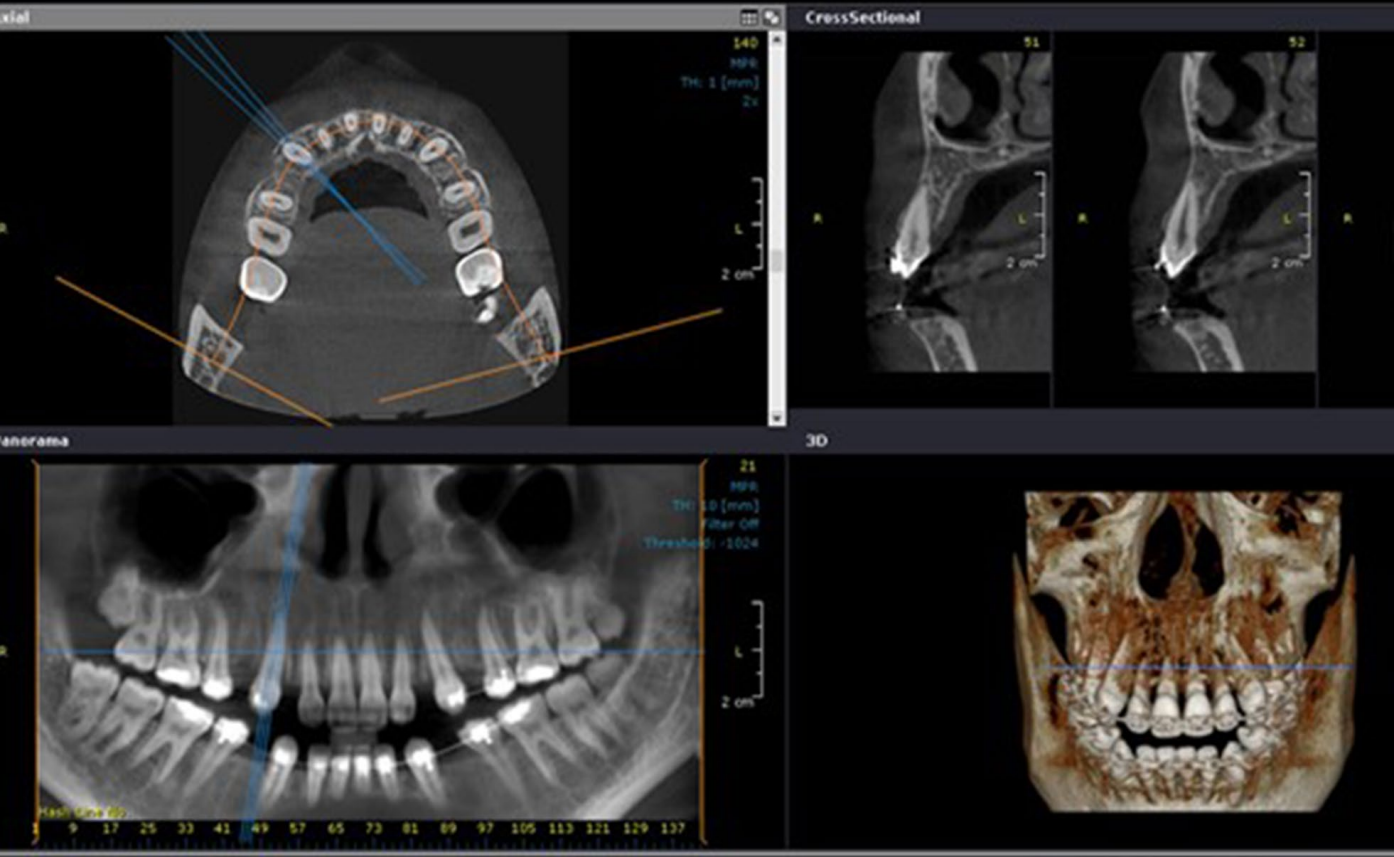
The arch section module of the OnDemand software, employed for the evaluation of canine root resorption

**Fig. 4:**
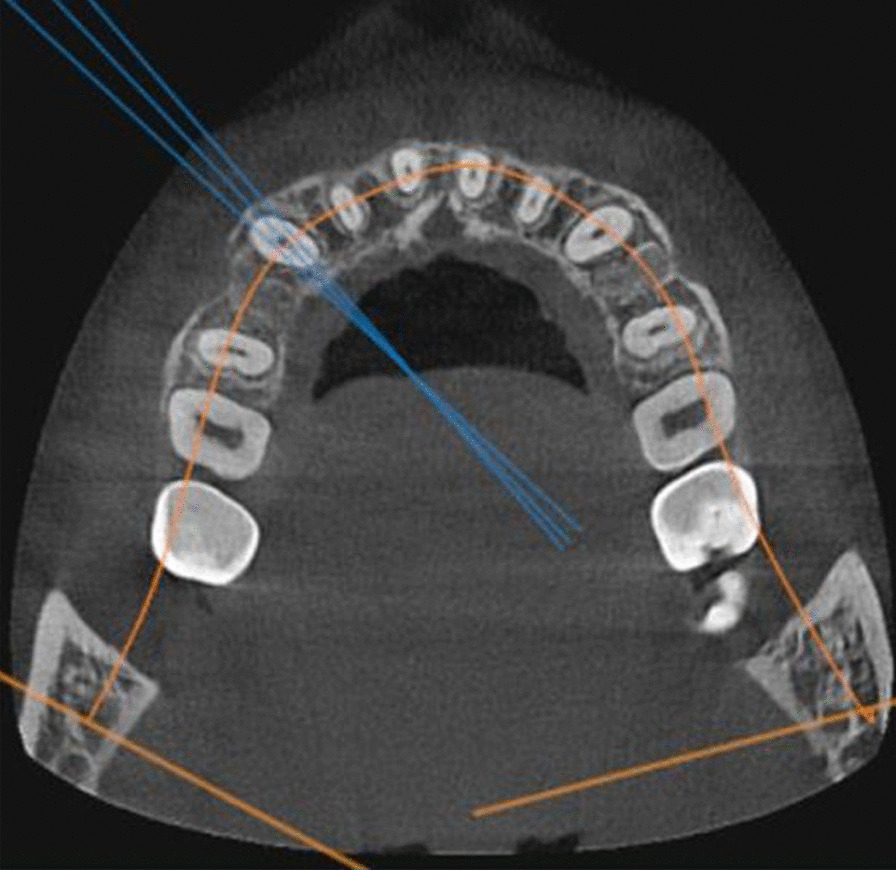
Axial view displaying the adjusted focal trough permitting labiolingual slicing of the maxillary canine on the right side, with an interval of 0.1 mm

**Fig. 5:**
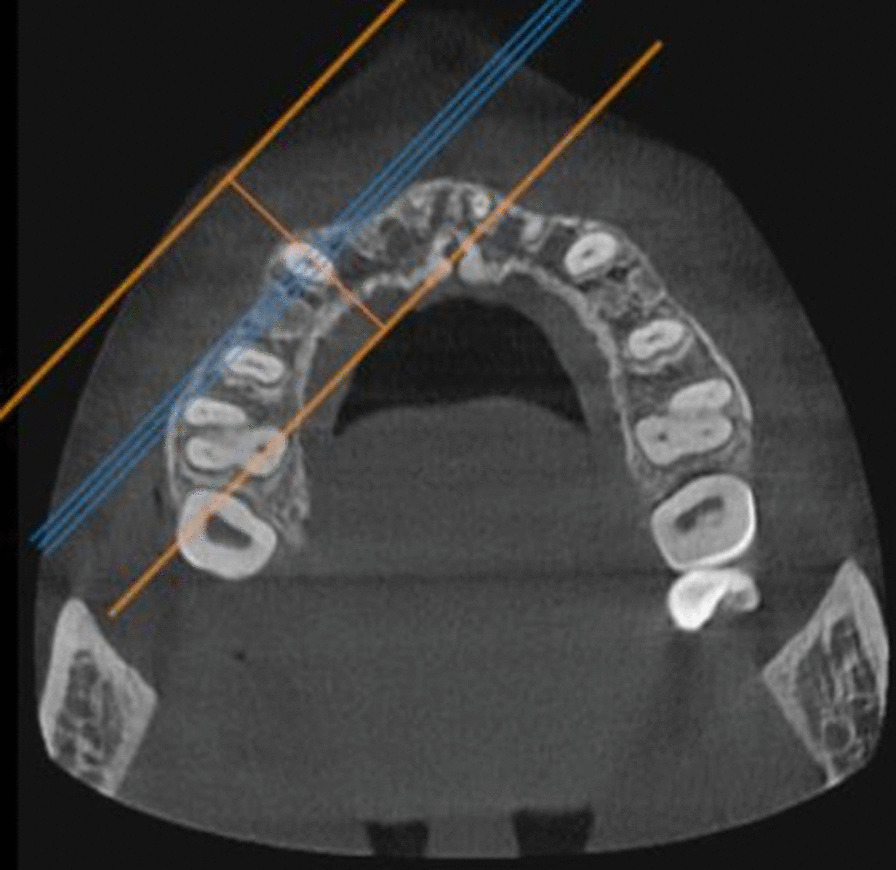
Axial view displaying the adjusted focal trough permitting mesiodistal slicing of the maxillary canine on the right side, with an interval of 0.1 mm

Using the software OnDemand3D**TM** (Cybermed Inc., South Korea), the pre- and post-retraction CBCTs obtained for each of the enrolled patients were utilized to assess the effect of the suggested two PBM protocols on canine root resorption, as follows:

1. The maxillary canines on the right and left sides were individually assessed in each CBCT.
2. Utilizing the arch section module, the focal trough was adjusted twice for each canine (Fig. 3). The first adjustment was to permit the labiolingual slicing/sectioning of the canine parallel to the long axis of its root (Fig. 4), whereas the second adjustment was to enable the mesiodistal slicing/sectioning of the canine, also parallel to the long axis of its root (Fig. 5). The least slice thickness interval was chosen, which was 0.1 mm.
3. The two perpendicular cross-sections showing the maximum length of the canine root were then selected for assessment using Malmgren index [32].
4. The right and left maxillary canines in each patient were operated upon as previously described, and each canine was given two scores from (0–4) in accordance with the degree of root resorption detected from the labiolingual as well as the mesiodistal cross-sections. These steps were performed for the pre- and post-retraction CBCTs.
5. The pre- and post-retraction scores for each canine were measured and evaluated statistically. The clinician was blinded to the experimental and control sides during measurement to avoid unwarranted bias. Measurements were also repeated by the same person one week later to check if there were measurement errors. Calibration of the root resorption measurements was performed, and intra-examiner reliability was calculated (Kappa = 0.96) [33], indicating excellent reliability.

### Statistical analysis

Frequencies and percentages were calculated for the root resorption scores. Comparisons of root resorption pre- and post-retraction scores between the two study groups were done using Mann–Whitney U test, while comparisons between the laser and control sides were done using Wilcoxon signed rank test. Comparisons between the root resorption scores pre- and post-retraction, within each group, were done using Wilcoxon signed rank test. Significance was set at *p* value < 0.05. Data were analyzed using IBM SPSS for Windows version 23.0 (IBM; Armonk, NY, USA).

## Results

Over the course of the study, there were no subject dropouts in the pre-intervention period, nor throughout the rest of the study. All the twenty initially recruited subjects completed the entire 12-week study period (10 subjects per group). Also, all the required CBCTs whether pre-retraction or post-retraction were accounted for. Demographic data and clinical characteristics of the enrolled subjects in both groups A and B, are presented in Table 1.

**Table 1:** Characteristics of the included study sample in both groups A and B

	Group A	Group B
Number of participants	10 subjects (n = 10)	10 subjects (n = 10)
Sex	Females	Females
Systemic condition	Healthy—no chronic diseases	Healthy—no chronic diseases
Previous orthodontic treatment	No previous orthodontic treatment	No previous orthodontic treatment
Periodontal condition	Healthy	Healthy

### The effect of PBM on OIIRR

Root resorption scores of the maxillary canines in groups A and B, on both the laser and control sides, are presented in Table 2. The roots of the maxillary canines were given scores according to Malmgren index as previously explained. In Group A, no changes in the pre-retraction and post-retraction root resorption scores have been recorded on the laser side. As for the control side, no patients were given a score of 2 in the pre-retraction records, but in the post-retraction numbers, two subjects were given a score of 2, comprising 20% of the group sample. However, this percentage change was not statistically significant.

**Table 2:** Comparison between the root resorption scores on the laser and control sides, pre- and post-retraction, in the two study groups

	Laser side	Control side	WSR *p* value
	N (%)		
Group A			
Pre-retraction			
Score 0	8 (80%)	7 (70%)	0.56
Score 1	2 (20%)	3 (30%)	
Score 2	0 (0%)	0 (0%)	
Score 3	0 (0%)	0 (0%)	
Post-retraction			
Score 0	8 (80%)	5 (50%)	0.10
Score 1	2 (20%)	3 (30%)	
Score 2	0 (0%)	2 (20%)	
Score 3	0 (0%)	0 (0%)	
WSR *p* value	1.00	0.06	
Group B			
Pre-retraction			
Score 0	8 (80%)	8 (80%)	1.00
Score 1	2 (20%)	2 (20%)	
Score 2	0 (0%)	0 (0%)	
Score 3	0 (0%)	0 (0%)	
Post-retraction			
Score 0	7 (70%)	6 (60%)	0.32
Score 1	3 (30%)	3 (30%)	
Score 2	0 (0%)	1 (10%)	
Score 3	0 (0%)	0 (0%)	
WSR *p* value	0.32	0.16	

*WSR* Wilcoxon signed rank test

In Group B, on the laser side, two patients were given a score of 1 in the pre-retraction measurements, comprising 20% of the group sample, but in the post-retraction records three patients were given a score of 1, thus accounting for 30% of the group sample. However, this change was insignificant statistically. For the control side, two patients were given a score of 1 pre-retraction (20% of the sample), while three patients were scored 1 in the post-retraction scores, accounting for 30% of the study sample, nevertheless this change was not statistically significant. Furthermore, no patients were given the score 2 in the pre-retraction measurements, yet one patient was given a score of 2 in the post-retraction records, representing 10% of the study sample, but this change also was not significant.

### Comparison between the root resorption scores on the laser sides in groups A and B

**Fig. 6:**
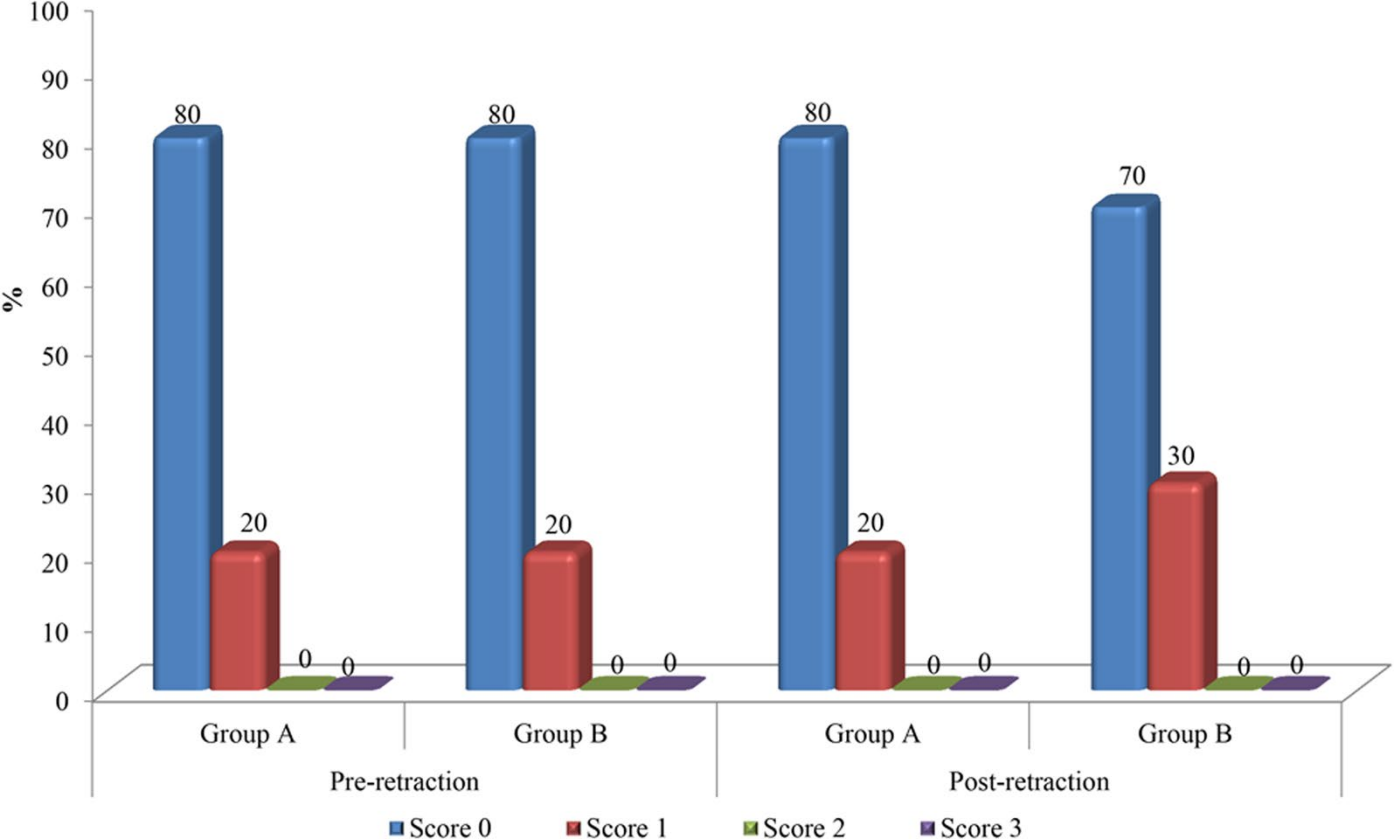
Root resorption scores on the laser sides of the two study groups, pre- and post-retraction

The difference in the canine root resorption pre- and post-retraction scores, on the laser sides of both groups A and B, is represented in Fig. 6. Between both study groups, no statistically significant differences have been recorded. In Group A, the laser side did not demonstrate any changes regarding the root resorption scores, pre- and post-retraction. As for Group B, a change of 10% was noted, where only two patients were given a score of 1 pre-retraction, and this changed to three patients with a score of 1 in the post-retraction records, which was an insignificant difference.

## Discussion

The aim of this study was to compare the effect of PBM on the OIIRR, using both the high frequency application protocol where laser has been applied on days 0, 3, 7, 14, and every 2 weeks thereafter (Group A), and the protocol with less patient recall, where laser application has been done at 3-week intervals (Group B). Both protocols have been documented in the literature [25, 26, 28, 30].

The current study design was a clinical randomized controlled trial (RCT). RCTs are contemplated as the gold standard for the evaluation of intervention efficacy [34]. The split-mouth technique also has been implemented, with its main advantage being the elimination of the inter-subject variability, as the patient acts and his/her own control, thus reducing the number of participants required.

One of the crucial factors influencing the therapeutic and biostimulatory effect of PBM is the dosage or energy density. In the current study, an energy density of 8 J/cm^2^ has been used, which is similar to that employed by Yousry et al. [35], as well as Goymen and Gulec [24]. By reviewing the literature, a wide range of energy density values have been documented with laser administration, where several authors used lower energy doses such as 3.6 J/cm^2^ [22], and 4.8 J/cm^2^ [16, 21], and others used higher doses such as 54 J/cm^2^ [36], and 75 J/cm^2^ [20]. In the present work, the administered laser energy dose was 8 J/cm^2^, delivered through a single application of 8 s against the maxillary canine root, dispensing a beam spot size of 1 cm^2^ using the flat top handpiece. A direct correlation has been documented between the beam size and the laser penetration depth, which in turn justifies the use of the flat top handpiece in this study [37, 38]. The same single application protocol with a large beam spot size was performed by Caccianiga et al. [39], and Abd El-Ghafour et al. [40].

The laser type employed in this study was a Diode laser semiconductor (Doctor Smile-Lambda Spa, Italy), used at a wavelength of 980 nm, as per manufacturer recommendation, in order to obtain the desirable bio-stimulatory effect. Generally, in the ultraviolet (UV) to the near infrared (IR) spectrum, the shorter wavelengths (200–600 nm) have more superficial penetration, in contrast to the longer absorption wavelengths (650–1200 nm) that have deeper tissue penetration [37]. The least penetrating wavelengths are in the far UV (excimer) and in the far IR (CO2) spectra, due to their high affinity to water [37]. Moreover, wavelengths in the 600–700 nm range are usually chosen for treating superficial tissues [41], whereas diode lasers in the near infrared ranges (810–980 nm) are selected for deeper-seated tissues, due to their longer optical penetration distances through the target tissues [41–43]. It has been also found that the near infrared diode lasers routinely used in dentistry, can reach a penetration depth of 4–5 mm into the target tissues, due to their poor absorption by water [43]. The 980 nm wavelength employed in the current study, has been used in several other studies where its biostimulatory effect has been advocated, such as that by Yassin et al. [21], where it has been reported that laser application can be effective in preventing, reducing, and repairing OIIRR. Also, in the study by Caccianiga et al. [39], the same laser device as that employed in our trial has been used, with the same 980 nm wavelength, and positive biostimulation has been reported. Effective biostimulation with the 980 nm wavelength has been also reported by Jivrajani and Bhad-Patil [44], as well as Abtahi et al. [45].

CBCT images were mandatory to investigate the effect of PBM on root resorption, thus pre- and post-retraction CBCTs (12-week interval) were performed by the patients enlisted in the study. Regarding CBCT measurements, high intra-observer and inter-observer reliability have been endorsed by El-Beialy et al. [46], and Tarazona-Àlvarez et al. [47]. Also, in comparison to periapical and panoramic X-rays, CBCT images have shown superior diagnostic precision in the detection of root resorption [48, 49]. Moreover, Malmgren index [32] was selected as a reliable scoring system for the assessment of root resorption. This assessment protocol has been adopted by other authors, such as Nimeri et al. [50], and Aboalnaga et al. [51].

Regarding the influence of PBM on OIIRR, according to the results of this study, no significant differences have been registered between the laser and control sides in both Group A and Group B. On comparing the root resorption values between the laser sides in both groups, using the different application protocols, also no significant differences have been reported. Therefore, it can be stated that according to our study results, PBM did not result in root resorption less than the commonly observed range, elicited with conventional orthodontic treatment, and that it has no effect on OIIRR.

However, controversial effects have been documented in the existent literature, regarding the impact of PBM on OIIRR. Similar results to those reported in the current study have been documented by Yousry et al. [35], and by Goymen and Gulec [24], and their results can be comparable to those of our study since they both also used an energy density of 8 J/cm^2^. Contrastingly, some researchers advocated the beneficial effects of employing PBM with OTM in reducing the expected associated root resorption, such as Ng et al. [52]. In the investigation by Ng et al. [52], four point laser irradiations have been conducted over the entire root length, starting from the root apex, followed by the middle centre of the root, and finally reaching the cervical third (on the mesial and distal sides), whereas in our study, the flat top handpiece was only held against the mid-root of the maxillary canine. The flat top handpiece employed in our study dispensed a large beam size of 1 cm^2^, and by holding it against the middle third of the root, the irradiation should reach the apical as well as the cervical parts. However, the apical third of the root did not receive direct laser irradiation, and this might explain the lack of a preventive or a reparative effect for PBM on OIIRR in the current study. Nevertheless, till now, the influence of PBM on root resorption is a rather disputable issue [53–55].

## Conclusions

With the parameters employed in this study, photobiomodulation does not affect orthodontically induced inflammatory root resorption, whether by increasing or decreasing its occurrence, with both laser application protocols. Therefore, it can be stated that PBM does not result in root resorption less than the commonly observed range elicited with conventional orthodontic treatment, and that it has no effect on OIIRR.

## Data Availability

The datasets used and/or analyzed during the current study are available from the corresponding author on reasonable request.
